# Determining the prevalence of Human Immunodeficiency Virus–Associated nephropathy (HIVAN) using proteinuria and ultrasound findings in a Nigerian paediatric HIV population

**Published:** 2012-01-22

**Authors:** Enobong Emmanuel Ikpeme, Udeme Ekpenyong Ekrikpo, Mkpouto Udeme Akpan, Samuel Itemobong Ekaidem

**Affiliations:** 1 Department of Paediatric Nephrology, University Of Uyo Teaching Hospital, Uyo, Nigeria; 2 Nephrology Unit, Department Of Internal Medicine, University Of Uyo Teaching Hospital, Uyo, Nigeria; 3 Department Of Chemical Pathology, University Of Uyo Teaching Hospital, Uyo, Nigeria

**Keywords:** HIVAN, microalbuminuria, HIV, HAART, proteinuria, paediatrics, Nigeria

## Abstract

**Background:**

HIV associated nephropathy (HIVAN) is the most common form of chronic kidney disease resulting directly from HIV infection. The true prevalence of HIVAN in the paediatric population of West Africa is unknown, largely due to lack of surveillance and reporting of kidney disease in HIV positive patients.

**Methods:**

This was a prospective study over a six month period( July to December 2008) conducted in the Infectious Disease Unit of the Department of Paediatrics, University of Uyo Teaching Hospital, Uyo, Nigeria involving all confirmed cases of paediatric HIV infection. Urine microalbuminuria using calculated urine albumin – creatinine ratio was determined and repeated in 4 weeks interval. CD4 count and renal ultrasonography was done for all the patients. The correlation of urine albumin – creatinine ratio with CD4 count, duration of treatment with highly active antiretroviral therapy (HAART) and association with clinical staging of the disease was also examined.

**Results:**

Fifty – nine (60.2%) were males, thirty – nine (39.8%) were females with male to female ratio of 1.5:1. The prevalence rate of 31.6% HIVAN was found, out of which 3.1% had abnormal ultrasound findings. There was a significant correlation between CD4 count and urine albumin – creatinine ratio (r=−0.22, p=0.03). There was no correlation between urine albumin – creatinine ratio and duration on HAART (r=−0.10, p=0.31).

**Conclusion:**

Screening for microalbuminuria is essential for the early diagnosis and treatment of HIVAN in this age group.

## Background

Human immunodeficiency Virus (HIV) infection is associated with protean manifestations, with the kidney being a common target. A variety of kidney disorders, acute or chronic may occur during the course of the infection [1]. Since the first reports of kidney disease associated with AIDS in 1984 and 1985 in the USA, there has been increasing evidence of kidney disease as a major complication of HIV infection [2–4]. Renal disease associated with HIV infection has a broad spectrum of clinical syndromes which include: acute tubular dysfunction with fluid and electrolyte abnormalities and/or renal failure caused by infections and nephrotoxic drugs, HIV associated nephropathy (HIVAN), immune mediated glomerulopathies (IgA nephropathy, lupus-like syndromes) and HIV – associated thrombotic mesangiopathies, including atypical forms of haemolytic uraemic syndromes [5–7].

HIVAN is the most common form of chronic kidney disease resulting directly from HIV infection [8]. Proteinuria serves as its first sign [9]. The true prevalence of HIVAN in Africa is unknown, largely due to lack of surveillance and reporting of kidney disease in HIV positive patients. This is particularly so in children since in many paediatric centres, renal biopsies are not performed regularly in HIV–infected patients even with persistent proteinuria [6–7,10–14]. In the USA, Strauss et al [7] and others [11–14] reported a prevalence of childhood HIVAN of approximately 10-15% with over 95% being African American children. Studies in Southern [15] and Western [16] Nigeria have reported the prevalence of proteinuria among HIV-infected children to be 18.8% and 20.5% respectively. There's no reported prevalence of HIVAN in the country.

Although the definitive diagnosis of HIVAN requires a histological examination of renal tissues, clinical criteria can strongly suggest HIVAN in children [14, 16]. This study was carried out in order to determine the prevalence of HIVAN in a tertiary institution in Nigeria using persistent proteinuria with renal ultrasound changes as the basis for diagnosis [18–20]. Its correlation with CD4 count, duration of treatment with Highly Active Antiretroviral Therapy (HAART) and association with clinical staging of the disease was also examined.

Early detection of HIVAN may be beneficial in evaluating early treatment and thereby preventing further disease progression to end stage renal disease, needing renal replacement therapy.

## Methods

This prospective study was conducted in the Infectious Disease Unit of the Department of Paediatrics, University of Uyo Teaching Hospital (UUTH), Uyo from July 2008 to December 2008. The hospital is the only tertiary and referral centre in Uyo, capital of Akwa Ibom State of Nigeria. It is also the main centre for Paediatric HIV care. It serves the 3.9 million population of the State.

All confirmed cases of HIV infection seen at the Infectious Disease Unit (IDU) during the study period were recruited into the study. Diagnosis was made by using the polymerase chain reaction for children who were less than eighteen months of age, or by antibody detection for those older than 18 months. Patients with urinary symptoms, acute febrile illness, diabetes mellitus and chronic renal disease were excluded from the study.

Demographic information (age and sex), mode of transmission of the infection, clinical staging of the disease according to WHO classification [21] and duration of treatment with HAART were obtained.

Mid-stream urine was collected from each patient and tested for proteinuria by dipstick using Combi 10 and then stored at −70°C until analysis. Urine microalbumin was measured using immune-turbidimetric method [22]. The reagent was purchased from Pharma-tec Petrochemical GnmbH, Germany. The sample was reacted with a specific antiserum to form precipitate whose turbidity was measured at 340nm using a UV spectrophotometer, UV-7804C, Sunny made by Bran Scientific and instrument company, England. The amount of complex formed was directly proportional to the amount of microalbumin in the sample. Urine creatinine was determined by a colourimetric, two points kinetic Jaffe's reaction method [23] using commercial diagnostic reagents kit made by fortress diagnostics Limited Antrim. The assay was based on the reaction of creatinine, in the 1 in 50 diluted urine sample with alkaline solution of picric acid to form a red complex whose absorbence was read at 495nm. The urine albumin: creatinine ratio was therefore calculated and expressed as µg albumin/mg creatinine. Values greater than 30µg albumin/mg creatinine were considered positive for microalbuminuria whereas values less than 30µg/mg creatinine were considered normal [18]. Repeat tests were performed for the patients with asymptomatic proteinuria within 4 weeks interval and they were regarded as having persistent proteinuria if the results were still positive.

Renal ultrasound was carried out on all the patients. The diagnosis of HIVAN was made based on the presence of persistent proteinuria in these patients and Overt HIV Nephropathy was defined as persistent proteinuria associated with renal ultrasound changes [18]. CD4 counts of these patients were also determined to see if there was any correlation with HIVAN. Data was analysed using SPSS 15 version and presented with simple tables. Correlation was by Pearson's coefficient.

Ethical clearance was obtained from the Ethical committee of the University of Uyo Teaching Hospital, Uyo, Nigeria.

## Results

**Figure 1:**
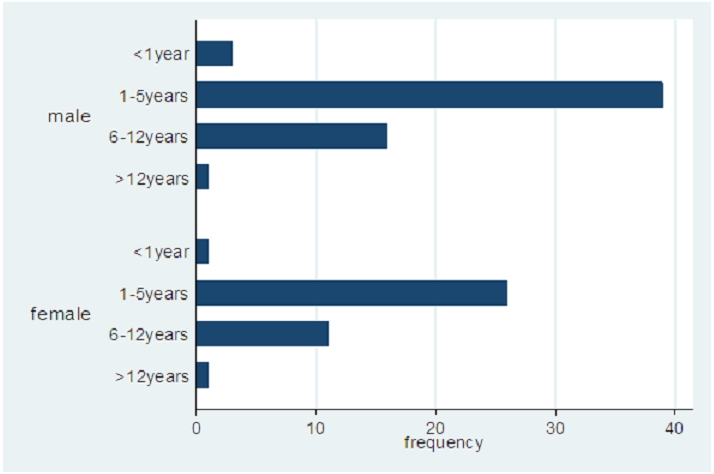
Age and gender distribution of the study population

Of the ninety – eight patients who met the selection criteria, sixty-five (66.3%) were aged 1 – 5 years, four (4.1%) were less than 1 year old, 27(27.6%) were aged 6 – 12 years while 2(2.0%) were older than 12 years. Fifty – nine (60.2%) were males, thirty – nine (39.8%) were females with male to female ratio of 1.5:1. Thirty-two patients (32.7%) had severe immunosuppression, 20(20.4%) moderate immunosuppression while 46(46.9%) had no evidence of immunosuppression using the immunological staging based on the CD4 count and the age of the patients (Ref). Fifty-seven (58.2%) were on highly active anti-retroviral therapy (HAART) for a varying period of time while 41(41.8%) were never on HAART. The age and gender distribution is shown in Figure 1.

There was no gender difference in the different variables sociodemographic and clinical characteristics of the patients (Table 1).

**Table 1:** Gender comparison of selected characteristics

Variable	Male	Female	P – Value
1 – 5 years	39 (66%)	26 (67%)	0.93*
CD4 count	772 (196 – 1170)	966 (409 – 1209)	0.27**
Duration on HAART (months)	30 (14 – 31)	23 (15 – 31)	0.28**
urine albumin-creatinine ratio (mg/g)	16.4 (8.1 – 35)	11.9 (7.8 – 30.7)	0.29**

* test of proportions

** Wilcoxon rank sum test

A total of 31 patients (31.6%) had HIV associated nephropathy (HIVAN) based on persistent proteinuria which was detected by either the dipstick method with urine protein of>1+ or urine microalbumin/creatinine ratio of>30mg/g repeated on two occasions of four weeks apart. Of these 31 patients with HIVAN, 28(28.6%) were diagnosed based on persistent proteinuria alone while 3(3.1%) were based on persistent proteinuria and abnormal ultrasound findings. The abnormal ultrasound findings were the presence of multiple hypoechoic foci in either one or both kidneys. This finding was common to the three patients. These patients had overt HIVAN.

All the patients had been on HAART for a median period of 27.5 months (interquartile range of 15 – 31 months) and the median CD4 count of the cohort was 813 cells/µL (interquartile range of 349 – 1196 cells/µL.

**Figure 2:**
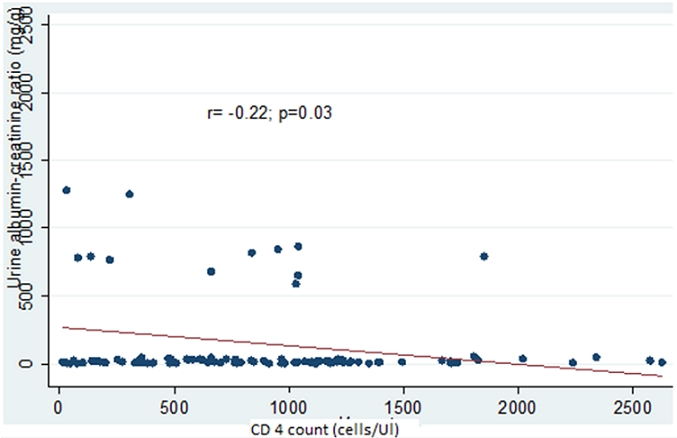
Plot showing the relationship between CD4 count and albumin excretion

**Figure 3:**
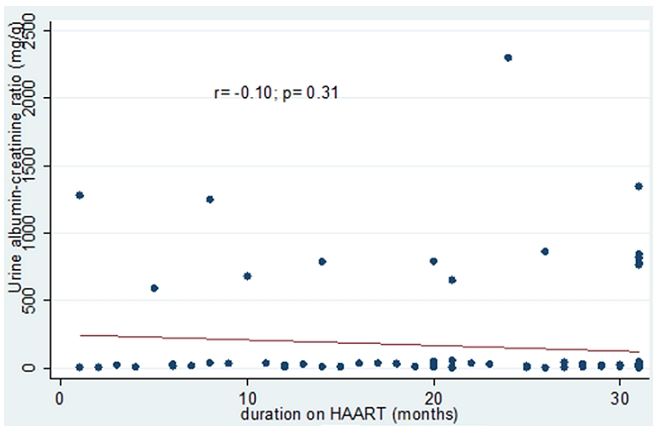
Plot showing the relationship between duration on Highly Active Anti-Retroviral Therapy (HAART) and albumin excretion

There was a significant negative correlation between CD4 count and levels of urine albumin-creatinine ratio (r= −0.22; P= 0.03). No correlation was found between the amounts of albumin excreted in urine and the duration the patient has been on HAART. These findings are depicted in Figure 2 and Figure 3.

## Discussion

HIV/AIDS pandemic continues to be a great health problem in Sub-Saharan Africa [22]. With the advent of highly active anti-retroviral therapy (HAART), more people are surviving the acute illness but are presenting with complications of the disease. The kidneys are not spared in this process. Kabanda et al [23] in Belgium noted that over 80% of HIV-infected patients without overt renal disease have evidence of glomerular permeability defects or tubular dysfunction, whatever the stage of the disease.

There are still very few reports of childhood HIVAN in Africa, despite the fact that it has a predilection for blacks with well over 90% of the patients from African descents [7, 11–14]. In this study, we report the prevalence of HIVAN in an African country. This study showed a male preponderance which is supported by other studies [18,24–25]. The development of HIVAN was not associated with any particular mode of transmission as reported in another study [26]. Vertical transmission was the major route of infection in this study as seen in other studies [9,14] although a previous Nigerian study reported a high rate of blood-transfusion acquired HIV infection. This disparity may be partly due to their studying already diagnosed children with HIVAN.

The prevalence of 31.6% we obtained compares favourably with the 24%19 and 29.8% [27] reported by some workers in South Africa and Washington DC respectively. Other workers however, reported lower prevalences of 19.4% [20] and 20.6% [18]. The relatively higher prevalence from this study may be attributable to the fact that all the patients in the study population were Africans. Overt HIVAN with ultrasonographic features of enlarged echogenic kidneys had a prevalence rate of 3.1%. This is significantly lower than 13.6% [18] reported by Chaparro et al. They had a larger sample size because their study was a 5 year retrospective chart review of patients.

Previously, some studies [14, 28] stated that HIVAN was a late manifestation of HIV infection. More recently other authors [18,24,30] have demonstrated HIVAN to be an early manifestation of HIV infection. This later report agrees with our findings. Only three of the thirty one patients with HIVAN presented in the late stages of the disease with severe immunosuppression as evidenced by very low CD4 counts below 100/mm^3^. However, Crowly and coworkers [31] did not report a correlation of HIVAN with viral load. This study was conducted in a very small population of 49 young men. This small sample size and gender bias would have resulted in this contrary conclusion. These three patients also had proteinuria in the nephrotic range. Proteinuria has been reported to be the first sign of HIVAN [9,14]. Other workers [19–20,27] noted microalbuminuria as an early manifestation of HIVAN as demonstrated in this study.

None of our patients presented with clinical features of peripheral oedema, hypertension and acute renal failure. This supports the reports of other authors [14, 26,30] that these are not common features of HIVAN. However, Anochie [24] in Nigeria reported these clinical features in the majority of her patients.

Bilateral echogenic kidneys that are often enlarged are common ultrasound findings in HIVAN, but normal size echogenic kidneys have been reported by some authors [3, 14,26, 30]. In this study, majority of our patients had normal ultrasonographic findings. The findings of enlarged hypoechoeic kidneys correlated significantly with severe immunosuppression and stages 3 and 4 disease.

HIVAN progresses to end-stage renal disease (ESRD) at a rapid rate, varying from weeks to months [32]. A fulminant course is seen in children compared with adults [33]. However, Ray et al [14] reported some impressive results with patients on HAART. Majority of our patients as at the time of conducting the study were already on HAART. Those that were new intakes were also commenced on HAART. As at the time of this report, none of them had developed renal symptoms, but are doing well on HAART, emphasizing the efficacy of HAART as a therapeutic measure for HIVAN. The HAART used is a combination of two nucleoside reverse transcriptase inhibitors (Lamivudine and Zidovudine) and one nonnucleoside reverse transcriptase inhibitor (Nevirapine). Other therapeutic measures include use of angiotensin converting enzyme (ACE) inhibitors and steroids in patients with renal manifestations and ESRD.

## Conclusion

In conclusion, the prevalence of childhood HIVAN in Nigeria is 31.6%. Screening for microalbuminuria is essential for the early diagnosis and treatment of HIVAN because severity of the disease correlates significantly with low CD4. The introduction of HAART has delayed the renal manifestations of the disease with subsequent progression to ESRD, but without substantial reduction in its prevalence.
